# Analysis of the Correlation between Occupational Accidents and Economic Factors in China

**DOI:** 10.3390/ijerph182010781

**Published:** 2021-10-14

**Authors:** Chengwu Li, Xiangbing Wang, Chengmin Wei, Min Hao, Zhen Qiao, Yonghang He

**Affiliations:** School of Emergency Management and Safety Engineering, China University of Mining and Technology (Beijing), Beijing 100083, China; lcw@cumtb.edu.cn (C.L.); cumtb_wxb@163.com (X.W.); safety19@126.com (Z.Q.); hyhaqxy@student.cumtb.edu.cn (Y.H.)

**Keywords:** occupational accidents, economic factors, correlation analysis, Gaussian grey model, accident statistics

## Abstract

One of the important factors affecting the production safety of a country or region is the level of economic development. Avoiding accidents under the condition of ensuring economic development is a problem that needs in-depth research. On the basis of collecting the data of occupational accidents and economic development indicators in China from 2000 to 2020, this paper studies the relationship between occupational accidents and five economic indicators, such as resident consumption, energy consumption, education funds, wage level and research input. The grey working accident model of Gaussian function is established, the occurrence trend of occupational accidents is quantitatively analyzed, and the accident reduction measures are suggested based on the relationship between accidents and economy. The results show that there is a strong correlation between accident and economic indicators, and the comprehensive correlation coefficient among scientific research investment, education funds and accident indicators is significantly higher than that of other economic indicators. Increasing investment in scientific research and education is conducive to improving the quality of workers and training safety professionals and can effectively reduce workplace accidents.

## 1. Introduction

In recent years, although China’s economy has made great progress, it is also faced with the problem of high number of accidents and deaths in production safety [1]. Although the government has taken many measures, including the reorganization of the General Administration of Safety Supervision, multiple rounds of amendments to the production Safety Law, and the continuous introduction of local and industrial safety standards [2], these simple administrative measures only alleviate the severe situation of production safety to a certain extent, and are not enough to completely solve the problem of production safety [3]. To investigate the reason, in addition to administrative means, the level of economic development is also one of the important factors affecting production safety. Rapid economic growth promotes the prosperity of the job market and enterprises accelerate production. However, the influx of a large number of untrained new labor force and loopholes in the enterprise safety management system will lead to a high incidence of occupational diseases and an increase in production safety accidents. Conversely, rapid economic development has led to the increase of investment in production safety, which can not only improve the level of staff training and education and the safety performance of production equipment, but also improve the level of production safety [4]. Therefore, the author considers that it is necessary to quantitatively analyze the correlation characteristics between production safety accident data and the level of economic development, as to provide help for the fundamental improvement of production safety situation and the rapid development of economy. Scholars from various countries have conducted considerable research on the relationship between economic growth and accidents and disasters. It can be divided into two aspects: the relationship between occupational safety and economic cycle and the relationship between industry accidents and economic development. There is an interdependent relationship between safety and economy, and safety cost is the link between safety and economy [5]. Based on this, Yeh [6] incorporated the economic cost of occupational accidents into the weighted data envelopment analysis (DEA) model to measure the safety and health economic performance of Taiwan’s industrial sector. Wei’s [7] research shows that socio-economic factors have a significant impact on occupational safety; the growth of socio-economic level can improve occupational safety, and occupational health and safety are related to the economic activities engaged in the country. Mallika [8] studied the economic evaluation of safety and summarizes the content and quality of the economic evaluation of injury prevention measures from 2010 to 2019. At the same time, economic indicators can be divided into macro- and microeconomic indicators, and there is a certain mathematical relationship between them and occupational injury rate. Tanya [9] compares the relationship between macroeconomic and microeconomic indicators and occupational injury rates in Western Australia, and studies show that economic cycle can have an impact on occupational injury rates. Farina’s [10] research is based on the linear autoregressive model, and this paper analyzes the correlation between Italian manufacturing injury rate and macroeconomic factors from 1994 to 2012. In addition, workplace accidents are an important economic phenomenon, and the recession is detrimental to workplace safety [11,12]. Béyszczarz [13] makes a regression analysis of Poland’s economic situation and occupational accident data during the period from 2002 to 2014, and the results show that the number of occupational accidents shows pro-cyclical behavior. Mouza’s [14] research is based on multiple regression analysis, and this paper studies the impact of common factors in the British economic cycle from 1971 to 2007 on fatal injuries. There is a strong correlation between the economic cycle and occupational accidents [15]. Beatriz [16] found that Spain’s economic growth is at the cost of high levels of occupational accidents. At the same time, economic development may lead to higher demand for occupational safety services, which may be more difficult for developing countries to meet [17]. In the field of fatal occupational accidents and national income, the Kahraman [18] study shows that for every 1% increase in national income, the incidence of fatal occupational accidents decreases by 1.1%. Moniruzzaman [19] found that the mortality rate of high-income countries first rose to a peak and then decreased with the improvement of the level of economic development. On this basis, Wu [20] extends the research method and uses the gradient lifting decision tree model to explore the joint influence of comprehensive factors on the number of accidents, casualties, economic losses and other indicators.

In addition, some researchers have carried out research on the relationship between accidents and economic development in different industries, including construction, mining, transportation and other industries. In the aspect of construction industry, Chen [21] and Hoyoga [22] established a multiple regression mathematical model to study the influence of socio-economic factors on building death toll and accident rate. Forteza [23] analyzed the relationship between accident rate in the construction industry and enterprise economy and found that there is a significant conic relationship between them. Xu [24] analyzed the important characteristics of fatal accidents in China’s construction industry from 2010 to 2019, such as accident type, severity and economic level. In the mining industry, Liu [25] found that large-scale mining of coal mines, metal and non-metal mines is an important factor in the high incidence of production safety accidents in our country. Tan’s [26] study confirmed that the lack of safety investments is one of the important reasons for the frequent occurrence of mining accidents in China, and increasing economic investment should be the key direction to improve the level of safety in production. At the same time, Dong [27] pointed out that an increase in equipment investment indicators can reduce fatal occupational injuries. In the transportation industry, socio-economic fluctuations will have a certain impact on road traffic accident indicators [28]. Paulozzi’s [29] research is based on per capita income and 100,000 mortality indicators, and this paper makes a statistical analysis of the relationship between economic development and traffic accident mortality in 44 countries. Sun [30] analyzed the impact of various economic factors on the number of traffic accident casualties in China, and the results show that economic development has a positive effect on improving traffic conditions. Rojo [31] and García [32] studied the correlation between road traffic accidents and real economic activities in Spain and analyzed the relationship between Spanish economic budget and road safety index. Law [33] used binomial regression to analyze the relationship between economic development and traffic accidents in 25 countries from 1970 to 1999 and proved that there is an inverted U-shaped curve relationship between economic development and traffic deaths. Li [34] and Bougueroua [35] analyzed the short-term and long-term causal relationship between the number of road traffic accidents and social and economic development and found that the number of traffic accidents was positively affected by per capita gross domestic product (GDP) in both the short term and long term. Traynor [36] made a regression analysis of per capita income and traffic accident mortality in Ohio and explained the impact of per capita income on traffic accident mortality.

Accordingly, socio-economic fluctuation is an important factor affecting accident disasters, which is closely related to accident mortality. Although scholars have carried out studies on the relationship between occupational safety, industry accidents and economic development, there are still deficiencies in the selection of economic indicators and quantitative analysis. This paper studied the characteristics of the evolution of the economy and accidents in China and constructed the index system of the relationship between the economy and accidents. In this paper, the influence of the economy on accidents is analyzed qualitatively and quantitatively, and a mathematical model is established to accurately describe the accident data. The conclusions of the study can provide a theoretical reference for the study of the factors affecting industrial accidents, the methods of controlling industrial accidents and the formulation of safety and economic policies in the future.

## 2. Materials and Methods

**Figure 1:**
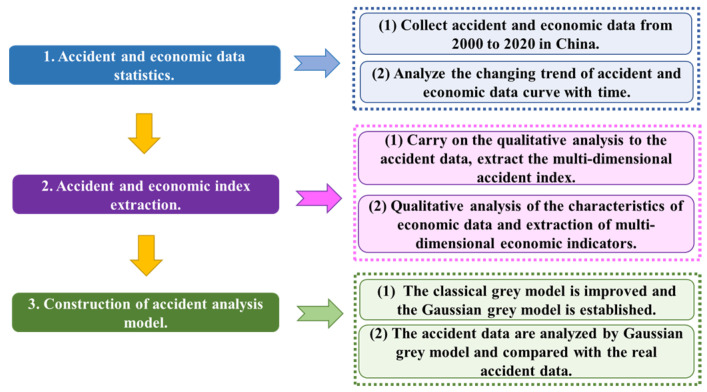
Flow chart of the research procedure.

The research materials are based on accident and economic data released by relevant Chinese government departments between 2000 and 2020. The above data are collated and analyzed, and the research methods and procedures are established, as shown in Figure 1. As can be seen from the figure, first of all, the accident and economic data are statistically studied, and their changing trends are analyzed. Second, in order to reflect the changing characteristics of accidents and the economy, multi-dimensional indicators of accidents and the economy are extracted based on the above statistical data. Finally, in order to quantitatively analyze the accident data, the traditional grey model is improved and a more optimized Gaussian grey model is established.

### 2.1. Changing Trend of Accidents and Economy

Economic development is the result of the comprehensive action of various social factors, which has a direct or indirect impact on production safety [37]. In the process of industrialization in developed countries, production safety has generally experienced a process from the rise and high incidence of accidents to a gradual and stable decline, and a correct understanding of the internal relationship and law between production safety and economic and social development [38]. It has important theoretical significance to guide the practice of production safety. At present, China is in a stage of rapid economic growth, accidents and disasters have become a common concern for the entire society [39]. Avoiding accidents under the condition of ensuring economic development is a problem that needs in-depth research. Therefore, the authors consider that it is necessary to strengthen the theoretical research on economic growth and accident disaster risk and to explore the relationship between accident disaster and economic growth. Through the Chinese Statistical Yearbook [40] and the Chinese Work Safety Yearbook [41], the data of accident deaths and gross domestic product (GDP) from 2000 to 2020 were collected, and the mortality rates of 100 million yuan and 100,000 workers were calculated. Among them, the mortality rate of 100 million yuan refers to the number of deaths caused by safety accidents in the process of producing 100 million yuan of GDP. The death rate of 100,000 people refers to the average number of deaths caused by accidents per 100,000 workers. The above statistical data were processed and drawn in the form of a time series, and the results are shown in Figure 1.

**Figure 2:**
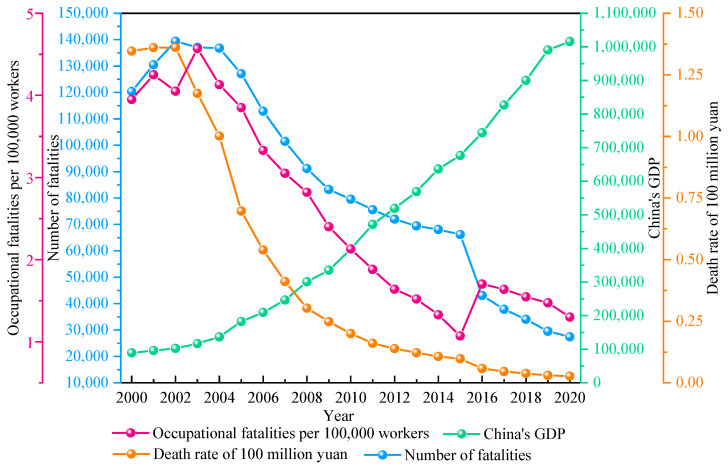
Changes in occupational accidents and economic data in China.

By analyzing Figure 2, we can find that China’s GDP shows an increasing trend from 2000 to 2020. Among them, the growth rate from 2000 to 2006 was slow, and from 2007 to 2019, it was significantly higher than before, and in 2020, due to the impact of the epidemic, the growth rate was slow. The number of accident deaths showed an increasing trend before 2002 and decreased year by year after 2002, especially in 2015–2016. The death rate of 100 million yuan was similar to that of accident deaths over time, which increased slowly before 2002, and then began to decline rapidly, and then slowed down significantly after 2016. The mortality rate of 100,000 workers showed a fluctuating upward trend from 2000 to 2004, decreasing rapidly year by year from 2004 to 2015, and declining slowly from 2016 to 2020. It can be concluded that there is a certain correlation between the number of deaths, 100 million yuan mortality and 100,000 deaths of the production safety accident index, and the GDP of the economic development index over time, but there are also differences. The safety system and the economic system are interrelated, showing a relationship of interdependence and mutual restriction. The level of disaster prevention and reduction in production safety and emergency management measures depends on the level of economic development; the various elements of safety development are restricted by economic level, and economic development is one of the fundamental causes of safety activities. The improvement of safety level relies on the support of economic development [42]. Conversely, safety in production also restricts economic development; the occurrence of accidents and disasters causes serious economic losses and brings a bad panic atmosphere to society, which brings resistance to the rapid and healthy development of the economy [43]. The authors consider that the current rapid development of China’s economy provides a guarantee for the sustained progress of safety, but the still relatively backward level of safety restricts economic development.

### 2.2. Construction of Index System of Accident and Economy

**Figure 3:**
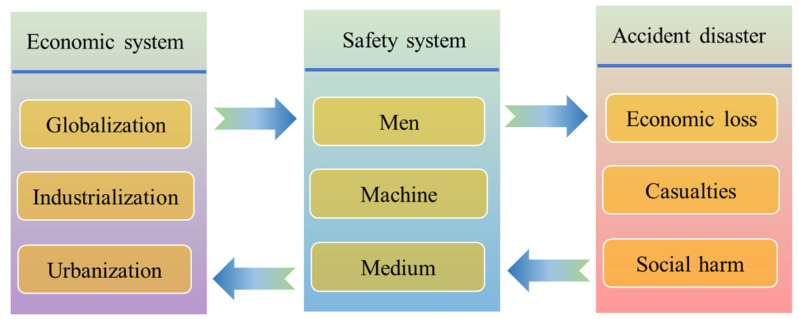
The correlation between safety incidents and economic development.

The safety system and economic growth are coupled with the social production system, and the accident disaster is the response of the imbalance of the social production system [44]. Therefore, the authors consider that the influence of the economic system on the accident disaster is indirect, and it is transmitted through the safety system. Among them, the economic system is composed of globalization, industrialization and urbanization modules; the safety system includes men, machine and medium modules; economic loss, casualties and social harm modules constitute the accident disaster, as shown in Figure 3. It can be found that with the development of globalization, industrialization and urbanization [45], the economic system presents an ever-changing and complex situation, which brings unmanageable pressure to a safe production environment. The complex relationship between safety elements is becoming increasingly difficult to handle [46,47]. For example, the development trend of urbanization is irresistible, the urban population and urban space are constantly increasing and expanding, the urban operation system is becoming increasingly complex, and the urban safety risks are constantly increasing. These hidden dangers, brought about by urbanization, have created immeasurable pressure on the original safety protection system, and there is a serious imbalance between the trend of rapid urban development and the ability of urban safety governance. The process of urbanization not only poses a severe test to the safety system [48], but also sets up invisible roadblocks for the safety development of a city.

Conversely, the rapid development of the economy has also brought huge investments in science and technology, talents, and funds for production safety [49]. These factors, which are beneficial to the development of safety, have brought a positive impact on production safety. From the perspective of a safety system, the confusion among safety elements will inevitably lead to future accidents and disasters. The occurrence of accidents and disasters cause economic losses, casualties and social harm, and these negative social effects are bound to destroy the normal production order and form constraints for economic development [50]. The restriction of production safety on economic development is becoming increasingly prominent, and the safety problem has gradually become an important prerequisite for normal operations of production. The safety system feeds back to the economic system through multiple angles, and its influence on the economic system continues to expand. Of course, the safety system with good toughness and flexibility and plays an important role in maintaining the rapid and stable development of the economy, and the adaptability of the safety system is the premise and guarantee of the stable operation of the economic system.

**Figure 4:**
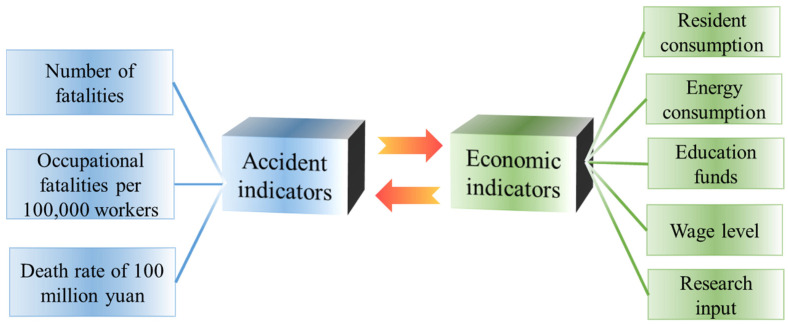
Characteristic system of correlation between accidents and economic indicators.

In order to reflect the changing characteristics of production safety accidents, the absolute index and relative index of accidents were extracted. The absolute index of accident indicates the number of deaths, and the relative index of accident indicates the mortality rate of 100 million yuan and 100,000 workers. For the description of the characteristics of economic development, the authors research is based on different levels of consideration, from the macro to the micro level and the national and individual levels. At the national level, the direct indicator to promote economic development is energy consumption, which provides supporting materials for economic development. Energy materials need to be transformed into productive forces, and the conversion efficiency is closely related to scientific and technological innovation, and the direct economic index of scientific and technological innovation is scientific research investment. At the personal level, it includes two dimensions: economic income and economic expenditure. The indicator of economic income is the wage level, which directly affects individual economic activities. The index of personal economic expenditure indicates resident consumption, and the level of resident consumption shows the situation of personal purchasing power. In addition, the comprehensive quality of individuals is related to safety [51], and the link between the national and individual levels is education, and the economic indicator of education is educational funds. To sum up, five dimensional indicators are extracted to measure residents’ consumption, energy consumption, education funds, average wages of employed personnel and scientific research expenditure. The characteristic system of the relationship between accident indicators and economic indicators is shown in Figure 4.

**Figure 5:**
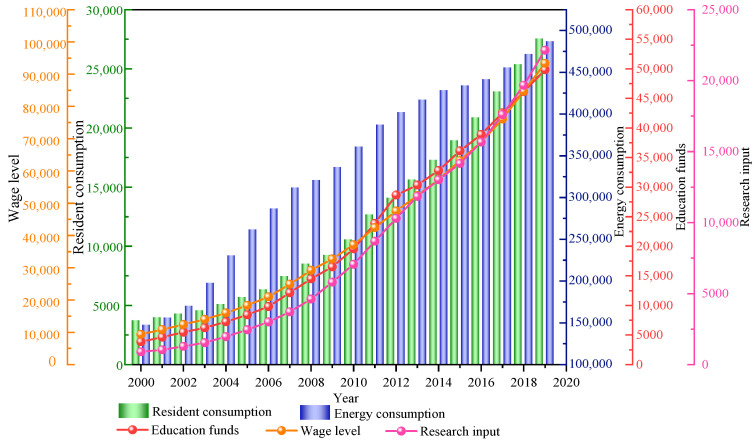
The changing trend of China’s economic indicators year by year.

The economic indicators in Figure 4 are explained in detail. The consumption level of residents refers to the sum of the expenditure used by consumers to meet their daily living expenses, which is equal to the total household consumption in the current year’s gross domestic product divided by the average population. Energy consumption refers to the sum of all energy consumed by the national production department in a certain period of time, including coal, oil, natural gas, primary electricity and other energy. Education funds are the expenses used by the state to develop education at all levels, including national financial education funds, investment by sponsors of private schools, social donation funds, career income and other education funds. Wage level refers to the average labor remuneration of employed persons in a certain period of time, which is equal to the total wages of employed personnel divided by the average number of employed people. Scientific research expenditure is the national expenditure for basic research, applied research and experimental development, including the labor fees of scientific research personnel, raw materials, the purchase and construction of fixed assets, management fees and other expenses. The above economic indicators are processed and plotted in the form of a time series, and the results are shown in Figure 5. Based on Figure 5, it can be seen that the five indicators of household consumption, energy consumption, education expenditure, average wage of employed personnel and scientific research expenditure show an increasing trend year by year, and the growth rate is more uniform and stable.

### 2.3. Accident Data Modeling and Prediction Analysis

In order to accurately predict the index data of production safety accidents, it is necessary to use a suitable mathematical model for research. The grey model has a good prediction effect for complex systems with uncertain factors, high prediction accuracy, and small sample data; thus, it can be used for short-term and medium- and long-term prediction of data [52,53]. However, only using the grey model avoids the mutual influence of the indexes, reduces the sensitivity of the more important indexes, and leads to the inaccurate prediction results [54]; thus, it is necessary to improve the model. Based on this, this paper uses the Gaussian function to further optimize the model and establishes the grey model of the Gaussian function (GGM(1,1)). In order to ensure the feasibility of the prediction model, it is necessary to check and process the known data. Let the original data be listed as *x*^(0)^ = (*x*^(0)^ (1), *x*^(0)^ (2), …. *x*^(0)^ (*n*)), which calculates the order ratio of the sequence as follows:

(1) $$\lambda (k)=\frac{x^{\left(0\right)}(k-1)}{x^{\left(0\right)}(k)},\;k=2,3,\ldots ,n$$

where *λ*(*k*) denotes the order ratio of the sequence, *x*^(0)^(*k* − 1) denotes the *k* − 1 term of the original data series, and *x*^(0)^(*k*) is the *k* term of the original data series. If all the grades fall within the allowable coverage interval *X* = (*e*^−2/(*n*+1)^, *e*^2/(*n*+1)^), then the sequence *x*^(0)^ can be predicted by establishing a model, otherwise the data need to be transformed properly. Here, we introduce the Gaussian function. The general form of the Gaussian function is as follows:

(2) $$f(x)=ae^{-\frac{{\left(x-b\right)}^{2}}{2c^{2}}}$$

In the formula, *a* is the height of the peak of the Gaussian curve, *b* is the coordinate of the center of the peak, and *c* is called standard variance, which represents the bell-shaped width of the Gaussian curve. Let *x*^(1)^(*k*) be the cumulative generating sequence of the sequence *x*^(0)^, and *z*^(1)^(*k*) generate the sequence for the adjacent value of the sequence *x*^(1)^ considering the Gaussian function.

(3) $$x^{\left(1\right)}(k)=\sum_{i=1}^{k}x^{\left(0\right)}(i),\;z^{\left(1\right)}(k)=\frac{1}{2}(\frac{k-\mu }{\sigma^{2}}x^{\left(1\right)}(k)+\frac{k-1-\mu }{\sigma^{2}}x^{\left(1\right)}(k-1))$$

where *μ* denotes the expectation of Gaussian distribution and *σ* denotes the standard variance of Gaussian distribution. Then, the grey differential equation of GGM(1, 1) is:

(4) $$x^{\left(0\right)}(k)+az^{\left(1\right)}(k)=\frac{b}{\sigma^{2}}(k-\frac{1}{2}-\mu )$$

where *x*^(0)^(*k*) = *x*^(1)^(*k*) − *x*^(1)^(*k −* 1) is called the grey derivative; *a* is called development coefficient; *z*^(1)^(*k*) is called whitening background value considering Gaussian function; *b* is called grey action amount; *k* = 2, 3, …, *n* is substituted into the above formula.

(5) $$\{\begin{array}{l} x^{\left(0\right)}(2)+az^{\left(1\right)}(2)=\frac{b}{\sigma^{2}}(2-\frac{1}{2}-\mu )\\ x^{\left(0\right)}(3)+az^{\left(1\right)}(3)=\frac{b}{\sigma^{2}}(3-\frac{1}{2}-\mu )\\ \cdots \\ x^{\left(0\right)}(n)+az^{\left(1\right)}(n)=\frac{b}{\sigma^{2}}(n-\frac{1}{2}-\mu ) \end{array}$$

Introduce the matrix vector notation:

(6) $$Y=\left[\begin{array}{c} x^{\left(0\right)}(2)\\ x^{\left(0\right)}(2)\\ \cdots \\ x^{\left(0\right)}(n) \end{array}\right]\;B=\left[\begin{array}{cc} -z^{\left(1\right)}(2) & (2-\frac{1}{2}-\mu )/\sigma^{2}\\ -z^{\left(1\right)}(3) & (3-\frac{1}{2}-\mu )/\sigma^{2}\\ \cdots & \cdots \\ -z^{\left(1\right)}(n) & (n-\frac{1}{2}-\mu )/\sigma^{2} \end{array}\right]$$

Then, the problem now is to find the values of *a* and *b*, and the values obtained by using the least square method are as follows:

(7) $$\left[\begin{array}{l} a\\ b \end{array}\right]={\left(B^{T}B\right)}^{-1}B^{T}Y$$

If *k* = 2, 3, …, *n* is regarded as a continuous variable *t*, then the previous *x*^(1)^ is regarded as a time *t* function; thus, the grey derivative *x*^(0)^(*k*) becomes the derivative of the continuous function d*x*^(1)^(*t*)/d*t*, and the whitening background value *z*^(1)^(*k*) corresponds to the derivative *x*^(1)^(*t*). Thus, the white differential equation of GGM(1, 1) is as follows:

(8) $$\frac{dx^{\left(1\right)}(t)}{dt}+a\frac{b-\mu }{\sigma^{2}}x^{\left(1\right)}(t)=b\frac{b-\mu }{\sigma^{2}}$$

By solving the equation and sorting out the transformation, the following results can be obtained:

(9) $${\hat{x}}^{\left(1\right)}(k)=(x^{\left(0\right)}(1)-\frac{b}{a})e^{-\frac{a{\left(1-\mu \right)}^{2}}{2\sigma^{2}}}e^{-\frac{a{\left(k-\mu \right)}^{2}}{2\sigma^{2}}}+\frac{b}{a},\;k=1,2,\ldots ,n$$

Equation (9) is a Gaussian grey GGM(1, 1) mathematical model. When *μ* = 0, *σ* = 1, the equation can be written as follows:

(10) $${\hat{x}}^{\left(1\right)}(k)=(x^{\left(0\right)}(1)-\frac{b}{a})e^{-\frac{a}{2}}e^{-\frac{ak^{2}}{2}}+\frac{b}{a},\;k=1,2,\ldots ,n$$

Equation (19) is a standard Gaussian grey GGM(1, 1) mathematical model, and the value is obtained accordingly.

(11) $${\hat{x}}^{\left(0\right)}(k)={\hat{x}}^{\left(1\right)}(k)-{\hat{x}}^{\left(1\right)}(k-1),\;k=1,2,\ldots ,n-1$$

The formula for calculating the average relative error is:

(12) $$\delta =\frac{1}{n}\sum_{i=1}^{n}\left|\frac{{\hat{x}}^{\left(0\right)}(i)-x^{\left(0\right)}(i)}{x^{\left(0\right)}(i)}\right|$$

The accident index data of the previous statistics are calculated according to the traditional grey model and the Gaussian grey model, and the results are shown in Table 1. In Table 1, Year is the time, ranging from 2000 to 2020; True Value is the actual number of accident deaths in each year; GM(1, 1) Predicted Value is the calculated value of accident deaths in each year of GM(1, 1) model; GM(1, 1) Relative Error is the difference between the calculated and actual accident deaths of GM(1, 1) model in each year divided by the actual value; GGM(1, 1) Predicted Value is the calculated value of accident deaths in each year of GGM(1, 1) model. GGM(1, 1) Relative Error is the difference between the calculated value and the actual value of the number of accident deaths in each year of the GM(1, 1) model divided by the actual value.

From Table 1, it can be found that the relative error of the GM(1, 1) model is large and the stationarity is poor, the maximum relative error is 0.28, and the relative error increases after 2014, and the prediction is relatively poor. Comparatively speaking, the overall relative error of GGM(1, 1) model is small and stable. The relative error is basically less than 0.05, which still has a good effect in the later stage of prediction. It can be shown that the GM(1, 1) model has a certain effect in describing accident data, but the effect of using GM(1, 1) model directly is not good. According to the characteristics of the data, this paper proposes an improved grey model, namely the Gaussian grey model GGM(1, 1), which is fitted and predicted by GGM model, and the corresponding estimated value is obtained. The method of solving the model parameters is simple and the relative error is small; thus, the Gaussian function grey GGM(1, 1) model can be selected to predict this kind of data. In addition, the traditional accident prediction model needs a lot of sample data, some of the models are subjective, and there are shortcomings, such as complex modeling, difficulty solving, etc. In this paper, the Gaussian function grey GGM(1, 1) can make full use of the sample data, removes the subjectivity in the modeling process, and has high accuracy. For the calculated data and equations, the independent variable of the calculated data is in the time range of 2000–2020, and the calculated equation is based on Equations (9) and (11), and the result is the number of deaths from accidents in each year. The formula can predict the number of accident deaths in the next few years. Through the use of this model, it is helpful to accurately predict the accident index and formulate a more reasonable safety policy, which is of great significance to ensure production safety and reduce accidents.

## 3. Results

In order to quantitatively analyze the correlation degree between different accident indicators and economic indicators, the grey correlation analysis method is used to measure it, and the correlation characteristic values are described based on grey correlation degree and grey absolute correlation degree. Grey relational analysis is a method based on grey theory to analyze the sequence of dynamic changes, and the correlation between them is judged by studying the correlation degree between indexes [55,56]. The advantage of this method is that when handling the system containing fuzzy information, it can deeply and accurately analyze the dynamic correlation between various factors. These factors are synchronously normalized and a mathematical model with correlation degree is established. The specific calculation steps of grey relational analysis are as follows:

First, the reference sequence and the comparison sequence need to be determined. The reference number is listed as the data sequence that reflects the behavior characteristics of the system, and the comparison number is listed as the data sequence composed of factors that affect the behavior of the system. The reference series can be expressed as:

(13) y={y(k)∣k=1,2,…,n}=(y(1),y(2),…,y(n))

If the influence factors of the reference sequence are *m*, the comparison sequence can be expressed as:

(14) $$x_{i}=\{x_{i}(k)|k=1,2,\ldots ,n\}=(x_{i}(1),x_{i}(2),\ldots ,x_{i}(n)),i=1,2,\ldots ,m$$

According to Equations (13) and (14), the initial incidence matrix composed of reference sequence and comparison sequence can be obtained as follows:

(15) $$y=\left\{\begin{array}{cccc} x_{1}(1) & x_{1}(2) & \ldots & x_{1}(n)\\ x_{2}(1) & x_{2}(2) & \ldots & x_{2}(n)\\ x_{m}(1) & x_{m}(2) & \ldots & x_{m}(n) \end{array}\right\}$$

Because of the different dimensions of the data in each factor column in the system, it is not easy to compare, or it is difficult to obtain the correct conclusion. Therefore, in the grey correlation degree analysis, it is generally necessary to handle the data dimensionless, and the processing method adopts the following equation:

(16) $${\tilde{x}}_{i}(k)=\frac{x_{i}(k)}{x_{1}(k)}$$

Therefore, the correlation between the comparison sequence *x_i_* and the reference sequence *y* can be expressed as follows:

(17) $$\xi_{i}(k)=\frac{\underset{i}{\min}\underset{k}{\min}|y(k)-x_{i}(k)|+\rho \underset{i}{\max}\underset{k}{\max}|y(k)-x_{i}(k)|}{\left|y(k)-x_{i}(k)|+\rho \underset{i}{\max}\underset{k}{\max}|y(k)-x_{i}(k)\right|}$$

Let $\Delta_{i}(k)=\left|y(k)-x_{i}(k)\right|$, then the Equation (17) is:

(18) $$\xi_{i}(k)=\frac{\underset{i}{\min}\underset{k}{\min}\Delta_{i}(k)+\rho \underset{i}{\max}\underset{k}{\max}\Delta_{i}(k)}{\Delta_{i}(k)+\rho \underset{i}{\max}\underset{k}{\max}\Delta_{i}(k)}$$

where *ξ_i_*(*k*) denotes the correlation degree between the comparison sequence and the reference sequence; *ρ* is the resolution coefficient, and the value interval of *ρ* is (0~1), usually the value of *ρ* is 0.5, where $\underset{i}{\min}\underset{k}{\min}\Delta_{i}(k)$ denotes the difference between the absolute minimum value of the factor of each comparison sequence and the factor of each reference sequence, and $\rho \underset{i}{\max}\underset{k}{\max}\Delta_{i}(k)$ is the difference of the absolute maximum value of the two. Because the correlation degree is the value of the correlation degree between the comparison series and the reference series at each time, and its value is more than one, the dispersion of information is not convenient for overall comparison. Therefore, it is necessary to concentrate the correlation coefficient of each time into a value. That is, to find its average, as a quantitative expression of the correlation degree between the comparison series and the reference series, and the solution equation is as follows:

(19) $$\gamma_{i}=\frac{1}{n}\sum_{k=1}^{n}\xi_{i}(k)$$

In order to improve the accuracy of the correlation coefficient, the Pearson correlation coefficient in statistics is coupled with the formula. The calculation formula of Pearson correlation coefficient is as follows:

(20) $$\varepsilon_{i}=\frac{\sum_{k=1}^{n}\left(x_{i}(k)-{\bar{x}}_{i}(k)\right)\left(y(k)-\bar{y}(k)\right)}{\sqrt{\sum_{k=1}^{n}{\left(x_{i}(k)-{\bar{x}}_{i}(k)\right)}^{2}}\sqrt{\sum_{k=1}^{n}{\left(y(k)-\bar{y}(k)\right)}^{2}}}$$

Based on the correlation degree and Pearson correlation coefficient, the calculation equation of comprehensive correlation coefficient is proposed as follows:

(21) $$\rho_{i}=\varphi \gamma_{i}+\left(1-\varphi \right)\varepsilon_{i}$$

where *ρ_i_* denotes a comprehensive correlation coefficient, which reflects not only the similarity between the comparison sequence and the reference sequence, but also the closeness of the change rate relative to the starting point. It is a quantitative index to comprehensively characterize whether the relationship between the sequences is close or not. *φ* is a proportional coefficient, usually 0.5. The accident and economic index data of the previous statistics are calculated according to Equations (19)–(21) respectively, and the results are shown in Table 2. In Table 2, *γ_i_* is the grey correlation degree, *ε_i_* is the Pearson correlation coefficient, *ρ_i_* is the comprehensive correlation coefficient, and the comprehensive accident index is the average correlation coefficient of the number of accident deaths, mortality of 100 million yuan and mortality per 100,000 workers.

**Figure 6:**
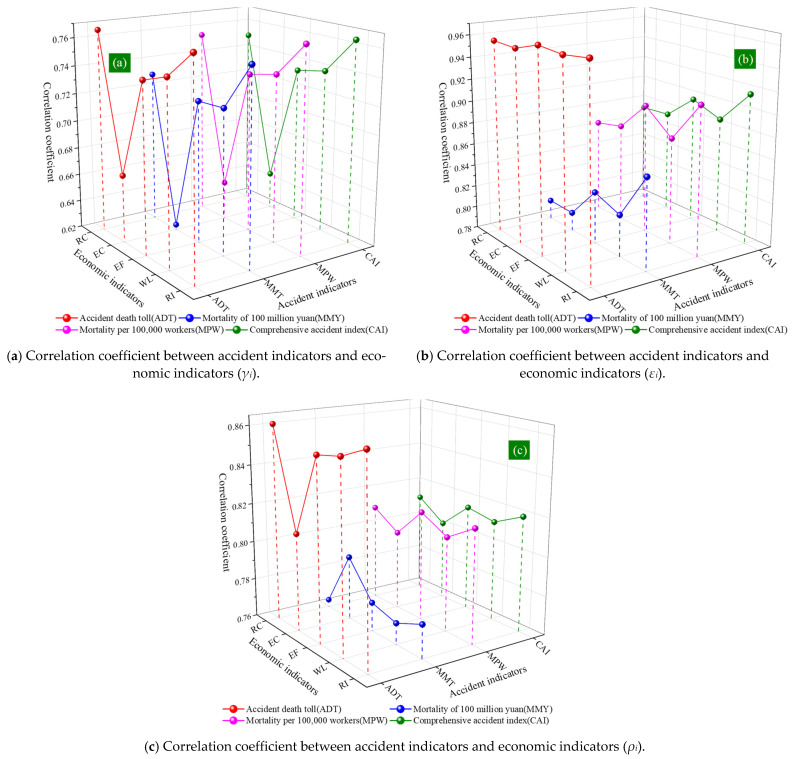
Different correlation coefficients between accident indicators and economic indicators.

The data in Table 2 is drawn as Figure 6. In Figure 6, the x direction is the economic index, corresponding to the abbreviations of the headings in the first row of Table 2; the y direction is the accident index, corresponding to the abbreviations of the headings of the first column in Table 2; the z direction is the correlation coefficient between the accident index and the economic index. Because 2–5 rows of data in Table 2 are grey correlation degree, 6–9 rows of data are Pearson correlation coefficients, and 10–13 behavior comprehensive correlation coefficients, they are plotted as (a), (b) and (c) graphs, respectively. (a) It can be found that under the condition of different accident indicators, the change trend of economic indicators is similar. The minimum correlation coefficient between the accident index and economic index is energy consumption, and the maximum value is scientific research investment. As can be seen from (b), the correlation coefficient of Accident death toll (ADT) is significantly higher than that of other accident indicators, and the overall value of mortality of 100 million yuan (MMY) is smaller. Under the condition of different accident indicators, the overall value of scientific research investment and education expenditure is higher, while the overall value of energy consumption is lower. Based on (c), it can be found that the overall correlation coefficient of ADT is higher, and the overall value of MMY is smaller. The overall value of scientific research investment and education expenditure is higher, the value of energy consumption is not stable, and the correlation coefficient with ADT is lower, but the correlation coefficient with MMY is higher.

## 4. Discussion

### 4.1. Analysis on the Characteristics of Accidents and Economic Indicators

**Figure 7:**
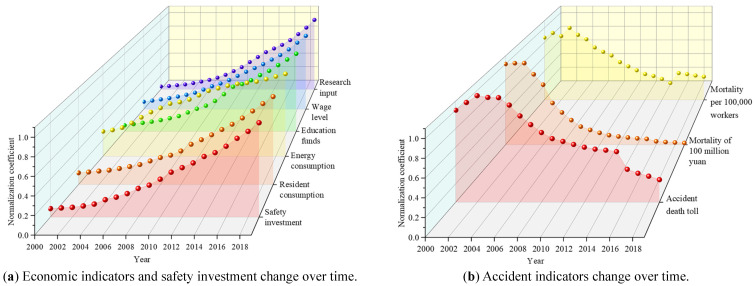
Economic indicators and accident indicators change with time.

Based on the China Statistical Yearbook [40] and the China production Safety Yearbook [41], the authors produced statistics on the safety investment data from 2000 to 2019. Due to the large differences in the values of safety investment, five economic indicators and accident indicators, they were normalized and drawn, as shown in Figure 7a,b.

Figure 7a shows the change curve of safety investment and five economic indicators with time. From the chart, we can see that safety investment and five economic indicators show an increasing trend with time, but the growth rates of different indicators are different. The growth rate of safety investment, household consumption, wage level and scientific research investment is relatively uniform, with slow growth in the early stage and fast growth in the later stage. The change rate of energy consumption and education funds has a large fluctuation but shows an increasing trend. Figure 7b shows the variation curve of three accident indexes with time. It can be seen from the chart that the three accident indexes basically show a downward trend with time, but the decline rates of different indicators are different. The number of accident deaths increased slightly in the early stage and gradually began to decline in the later stage, falling rapidly from 2016. The mortality rate of 100 million yuan decreased slowly from 2000 to 2002 and slowed down with the increase of the decline rate. The mortality rate of one million workers fluctuated from 2000 to 2002, began to decline in 2003, increased slightly in 2016, and then continued to decline. Compared with Figure 7a,b, safety investment, five economic indicators and accident indicators basically show a negative correlation, indicating that increasing safety investment can effectively reduce accident mortality. The five economic indicators and safety investment are discussed in detail.

Energy consumption indicators profoundly reflect the industrial structure of a country, and the degree of optimization of industrial structure in turn determines the total amount of energy consumption and the level of utilization. Accident disasters are closely related to industrial structure, and the risk intensity of industrial accidents in the secondary industry, such as construction and mining, is higher than that in the primary and tertiary industries [57,58]. Therefore, the authors believe that in the economic structure of a country or region, the higher the proportion of non-agricultural industries in the three industrial structures, the greater the pressure of accidents and disasters. The transfer of employment from more dangerous industries to industries with lower accident disaster risk will reduce the accident disaster risk of the society as a whole. With the decline of the proportion of employment in the secondary industry, more employees flow into safer industries, which may reduce the disaster risk of industrial accidents in a country or region. Therefore, the analysis of the changing process of the industrial sector is of key significance to understand the macro-characteristics of the evolution of accidents and disasters.

The two economic indicators of resident consumption and wage level correspond to human capital in occupational safety. Accidents may bring life or health risks to people in the operating system, or lose their lives, cause the loss of human resources, reduce the quality and quantity of human capital, and thus have a negative impact on economic growth. Conversely, organizations create value by converting inputs into outputs through an operating system [59,60]. Enterprises accept the input of people, equipment and materials, and then convert them into goods or services that can meet the needs, and the realization of this goal cannot be achieved without a safe and stable production environment. In the production process, factors such as hardware instability and incoordination between human behavior and hardware or environment will disturb the system behavior and make it fluctuate. Once the magnitude of the fluctuation exceeds the safety capacity of the system, the production process will be interrupted. As a result, the accident occurs. Therefore, the quality of human capital not only directly affects production efficiency and economic growth, but also directly affects production safety.

Education funds and research input are closely related to the quality level of technical equipment and personnel and are related to the progress of safety science and technology. The role of safety science and technology on production safety is omni-directional, and it has an effect on people, machines, environment, management and other elements that constitute safety system engineering. Safety science and technology can enhance the technical reliability of the production system by improving the safety skills of workers, the safety and reliability of labor tools, the awareness of potential dangers of labor objects and the level of safety management, and reduce the environmental risks caused by accidents [61]. On the contrary, the fault of safety technology increases the risk of safety in production. The skills of human resources, including production workers, engineers and managers, and the ability to learn and understand technical materials are the key factors for technological innovation and reducing the risk of technology transfer. Only when the operators in the front line of production accurately operate, use the machinery and equipment, and have the ability and consciousness to control the potential dangers of mechanical work, can advanced science and technology be applied in production, reducing the unsafe behavior of people in the production process, and reducing the occurrence of production accidents.

Safety investment is closely related to five economic indicators, which refers to the sum of safety-related expenses spent by a country or enterprise, including safety measures, personal protective equipment, occupational disease prevention, etc. Safety investment is a direct consumption of various resources to improve safety, including safety equipment costs, accident handling costs, compensation, maintenance costs, etc. Conversely, it is the resources invested by improving the quality of personnel and the ability of safety prevention, including safety education, safety training, safety culture construction and so on. Increasing safety investment can continuously improve the quality of employees, improve the level of safety science and technology and safety management, and then reduce and control the unsafe behavior of people and the unsafe state of things, and achieve the purpose of safe production.

### 4.2. Research on the Change of Accident and Economic Indicators

**Figure 8:**
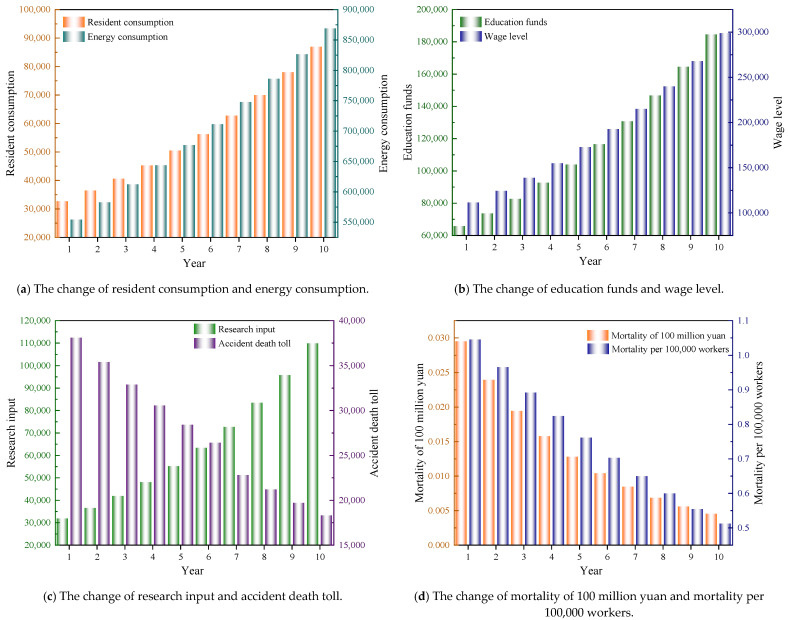
The change of economic indicators and accident indicators.

The previous section improved the grey model and established the Gaussian function model. Then, based on the Gaussian function model, the dynamic characteristics of economic indicators and accident indicators in the next ten years were analyzed, and the calculated results of the model were drawn as shown in Figure 8.

The analysis of Figure 8a shows that household consumption and energy consumption show an upward trend with time. It is calculated that the ten-year growth rate of household consumption is 1.272 times, and the ten-year growth rate of energy consumption is 0.569 times; thus, the overall growth rate of household consumption is faster than that of energy consumption. China’s energy consumption structure has been further optimized, and the consumption level of residents has improved significantly. Figure 8b shows the dynamic change of education expenditure and wage level. It is calculated that the ten-year growth rate of education expenditure is 1.518 times, and the ten-year wage growth rate is 1.393 times. The further increase of education funds will help to improve the overall quality of safety practitioners to reduce safety risks. The analysis of Figure 8c shows that the investment in scientific research shows a significant increase, while the accident mortality rate is gradually decreasing. It is calculated that the ten-year growth rate of scientific research investment is 1.865 times, and the accident mortality rate is 0.519 times. The significant increase in scientific research investment promotes the progress of safety science and technology and the improvement of the level of safety management and improves the risk prevention level of the safety system. Figure 8d shows the dynamic change of the mortality rate of 100 million yuan and one million workers. It is calculated that the ten-year reduction rate of 100 million yuan mortality is 0.847 times and that of million workers is 0.511 times, indicating the continuous improvement of production safety in China. The overall analysis of Figure 8 shows that based on the calculation results of the Gaussian function model; the five economic indicators of our country will further improve in the future. Among them, the growth rate of scientific research investment and education expenditure is relatively high, the growth rate of energy consumption is relatively low, and the growth rate of household consumption and wages is in the middle. The accident mortality rate is also gradually decreasing. The average accident mortality rate is divided by the average rising rate of economic indicators, and the calculated result is 0.472, indicating that the accident decline rate is slower than the economic growth rate.

### 4.3. Accident Reduction Measures Based on the Relationship between Accident and Economy

Based on previous research, there is a complex interaction between accident disasters and economic growth. The solution of safety problems and the coordination of production safety and economic growth need to be solved through the coordinated arrangement of macroeconomic development and safety policies at the national level. If the safety policy is separated from the economic policy, it will affect the implementation effect of the policy. Based on the mechanism of the relationship between accidents and economy, the following accident prevention and mitigation measures are proposed.

(1) Closely integrate the linkage policy mechanism of accident prevention and economic development. In the process of national economic development, it is especially necessary to strengthen the supervision of production safety and the risk monitoring of enterprise organizations to effectively resolve the negative impact of changes in the economic system and other socio-economic factors on workplace safety. Accident prevention needs to not only consider the factors of technology and personnel; the influence of economic factors cannot be ignored; thus, it is necessary to establish a comprehensive accident prevention mechanism with the integration of technology, personnel and economy. (2) Designing dynamic safety evaluation index in economic development. In the process of economic development, the government will involve the problem of technology transfer in different regions; thus, it is necessary to evaluate the safety and reliability of the technology transfer. Dynamic safety evaluation indicators considering economic factors should be designed to promote the diversification and dynamic of safety indicators, not limited to accident mortality. At the same time, we need to consider the economic differences of different regions and formulate safety policies in accordance with local conditions. (3) Increase the investment in education and scientific research to improve the quality of safety science and technology and safety personnel. Give full play to the driving force of science and technology to promote safety and enhance the efficiency of enterprises and strengthen basic and applied research on safety. Make full use of the research capacity of university-level scientific research institutions to establish and improve the safety service system of experts and intermediaries. According to the characteristics of the accident, we should conduct research on key technologies, effectively apply the achievements of safety scientific research, and improve the standard system of safety science and technology. (4) Optimize and adjust the industrial structure based on the law of the relationship between accidents and economy. We will speed up the adjustment of the industrial structure, give priority to the development of new energy-saving industries, and reduce the reliance on backward industries. At the same time, we should strictly implement the access system for backward industries, ensure safe investment from the source, and speed up the elimination of backward production capacity.

## 5. Conclusions

In this paper, the relationship between occupational accidents and economic factors is analyzed in detail. This paper constructs the correlation characteristic system of multi-dimensional economic indicators (resident consumption, energy consumption, education funds, wage level and research input) and accident indicators (accident death toll, mortality of 100 million yuan and mortality per 100,000 workers). The grey model is improved, the Gaussian function model is established, and disaster reduction measures are proposed based on the correlation mechanism between accidents and the economy. The main conclusions are as follows:

(1) There is a strong correlation between the accident index and economic index, and the correlation coefficient of different indexes is between 0.624 and 0.963. The correlation coefficients between different accident indicators and economic indicators are different, from large to small are scientific research investment, education funds, resident wages, resident consumption and energy consumption. Among them, the comprehensive correlation coefficient between scientific research investment, education funds and accidents is significantly higher than other economic indicators.

(2) The Gaussian function is incorporated into the grey model, and the Gaussian function model is established. Compared with the real accident data, the relative error of the Gaussian function model is less than 6%, which is lower than the maximum relative error of 15% of the grey model. The results show that the Gaussian function model has high accuracy and can be used for the calculation and analysis of accident data. In addition, the calculated data has high stability, and the method of solving the model parameters is relatively simple, which enriches the accident calculation model.

(3) The changing characteristics of the accident index and economic index with time are different. According to the calculation results of the model, there is an indirect negative correlation between the economic index and accident index. The growth rate of scientific research investment and education expenditure is relatively high, the growth rate of energy consumption is relatively low, and the growth rate of household consumption and wages is in the middle. The ratio of the average rate of accident decline to the rate of economic growth is 0.472. The research shows that in the process of formulating safety policy, we should consider the influence of economic factors and establish a comprehensive accident prevention mechanism with the integration of safety technology, personnel and the economy.

## Figures and Tables

**Table 1:** Predictive values and errors of accident data by different models.

Year	True Value	GM(1, 1) Predicted Value	GM(1, 1) Relative Error	GGM(1, 1) Predicted Value	GGM(1, 1) Relative Error
2000	120,351	120,350.239	−0.000006	120,398.833	−0.000037
2001	130,491	153,846.738	0.151812	130,893.063	−0.003072
2002	139,393	142,948.842	0.024875	137,745.544	0.011960
2003	137,070	132,822.911	–0.031976	139,394.868	−0.016678
2004	136,755	123,414.260	−0.108097	135,349.481	0.010384
2005	127,089	114,672.081	−0.108282	126,393.027	0.005506
2006	112,879	106,549.164	−0.059408	114,390.916	−0.013217
2007	101,480	99,001.642	−0.025034	101,753.681	−0.002690
2008	91,172	91,988.756	0.008879	90,729.347	0.004879
2009	83,200	85,472.636	0.026589	82,756.006	0.005365
2010	79,552	79,418.092	−0.001686	78,086.517	0.018767
2011	75,572	73,792.428	−0.024116	75,806.112	−0.003088
2012	71,983	68,565.264	−0.049846	74,231.713	−0.030293
2013	69,434	63,708.371	−0.089872	71,554.045	−0.029629
2014	68,061	59,195.522	−0.149766	66,503.627	0.023418
2015	66,182	55,002.345	−0.203258	63,816.640	0.037065
2016	43,062	51,106.196	0.157402	44,347.675	−0.028991
2017	37,852	47,486.035	0.202881	39,798.570	−0.048911
2018	34,046	44,122.311	0.228372	32,863.764	0.035974
2019	29,519	40,996.861	0.279969	29,087.441	0.014837
2020	27,412	38,092.805	0.280389	27,954.130	−0.019394

**Table 2:** Correlation coefficient between accident indicators and economic indicators.

	Resident Consumption	Energy Consumption	Education Funds	Wage Level	Research Input
1. Accident death toll (*γ_i_*)	0.766539	0.669142	0.742253	0.749791	0.769856
2. Mortality of 100 million yuan (*γ_i_*)	0.729998	0.623648	0.722355	0.723761	0.757859
3. Mortality per 100,000 workers (*γ_i_*)	0.75478	0.647696	0.735713	0.741025	0.766227
4. Comprehensive accident index (*γ_i_*)	0.750439	0.646829	0.73344	0.738192	0.764647
5. Accident death toll (*εi*)	0.956225	0.955454	0.963937	0.962162	0.963653
6. Mortality of 100 million yuan (*ε_i_*)	0.797445	0.79676	0.827531	0.818164	0.862573
7. Mortality per 100,000 workers (*ε_i_*)	0.865659	0.870746	0.898157	0.876959	0.914696
8. Comprehensive accident index (*ε_i_*)	0.87311	0.87432	0.896542	0.885761	0.916307
9. Accident death toll (*ρ_i_*)	0.861382	0.810398	0.853095	0.855976	0.862655
10. Mortality of 100 million yuan (*ρ_i_*)	0.763721	0.79311	0.774943	0.770962	0.777309
11. Mortality per 100,000 workers (*ρ_i_*)	0.81022	0.801196	0.816935	0.808992	0.818486
12. Comprehensive accident index (*ρ_i_*)	0.811774	0.801568	0.814991	0.811977	0.819484
